# A Qualitative Analysis of the Experiences of Young Patients and Caregivers Confronting Pediatric and Adolescent Oncology Diagnosis

**DOI:** 10.3390/ijerph20146327

**Published:** 2023-07-08

**Authors:** Ines Testoni, Anna Elena Nicoletti, Matilde Moscato, Ciro De Vincenzo

**Affiliations:** 1 Department of Philosophy, Sociology, Education and Applied Psychology (FISPPA), University of Padova, 35131 Padua, Italy; ciro.devincenzo@unipd.it; 2 Sagol Creative Arts Therapies Research Center, Haifa 31905, Israel; annae.nicoletti14@gmail.com (A.E.N.); moscato.matilde@gmail.com (M.M.)

**Keywords:** pediatric oncology, adolescent oncology, caregivers’ burden, qualitative research, psychological stress, personal growth

## Abstract

Epidemiological studies show that new cases of young oncology patients are increasing by 400,000 every year. Psychological literature has shown that receiving an oncological diagnosis can cause significant psychological stress and discomfort. However, the experiences of young patients and their caregivers as they confront this challenge are not yet fully understood. This paper adopts a qualitative methodological approach to explore how young patients with an oncological diagnosis and their parents make sense of the experiential challenges they face. Thus, the research realized 18 semi-structured interviews, 11 of which were with pediatric and adolescent oncology patients, and 7 of which were with 6 mothers and 1 father. The qualitative thematic analysis revealed that the oncological diagnosis triggers different emotions taking the scene in the attempt to cope with the threats of meaning that the diagnosis poses. However, such intense experience promotes transformative feelings in parents and young patients, leading to important personal growth. Lastly, this article discusses the need to improve palliative psychological care competences in pediatric oncology. By providing comprehensive psychological care to young oncology patients and their families, healthcare providers can mitigate the psychological stress and pain associated with the diagnosis and treatment of cancer.

## 1. Introduction

Every year, approximately 400,000 new cases of cancer are diagnosed in children and adolescents worldwide [1]. This trend is expected to continue increasing compared to the previous decade [2]. Pediatric oncology refers to all oncological pathologies that affect children and adolescents between 0 and 19 years old [3]. The survival rate for children and adolescents living in high-income countries is around 80% five years after diagnosis [4]. Cancer is a leading cause of non-traumatic death among young patients worldwide, and its incidence is expected to continue increasing in the coming years [5]. In Italy, where this research was conducted, a study by the Italian Association of Tumor Registries [6] found that 7000 neoplasms were diagnosed among children and 4000 among adolescents (15–19 years) between 2016 and 2020, consistent with diagnoses recorded between 2011 and 2015. The estimated annual incidence is approximately 1400 cases for children (0–14 years) and 900 for adolescents (15–19 years).

In addition to the medical consequences and concerns for young patients, there are also psychological, ethical and social concerns [7]. Focusing on the individual, children and adolescents in active treatment experience significant changes in their bodies, which greatly influence their daily lives and emphasize their desire to feel “normal” [8]. Among the most common physical symptoms are pain and fatigue [9], as well as changes in motor and respiratory functioning, difficulty swallowing and nausea and vomiting. From a psychosocial perspective, these young patients often experience strong feelings of anxiety, depression, fear, low self-esteem, social and school difficulties, irritability, nervousness, sadness and sleep problems [10,11].

Several studies have highlighted the primary and secondary needs of pediatric and adolescent patients affected by different types of neoplasms. Primary needs include the desire to receive love, respect and protection of one’s dignity; the need to have caregivers close, both emotionally and physically; the desire to be personally involved in the care, treatment and decisions to be made; and the need to maintain a positive relationship with healthcare staff [12]. Secondary needs include the need to openly communicate one’s emotions, to be listened to without judgment and to maintain, as much as possible, one’s independence, while still acknowledging the limitations imposed by the disease [13].

Effective pediatric palliative care requires a collaborative approach between healthcare professionals, family members and patients, which addresses not only the medical needs but also the social, spiritual and psychological aspects of the child and family [14]. Moreover, children and adolescents in active treatment struggle to make sense of their illness and may feel that they are missing out on important moments in life [15]. Adolescents diagnosed with cancer and undergoing more intensive treatments tend to have worse psychosocial outcomes and lower quality of life, while those diagnosed with cancer during childhood often demonstrate psychological resilience, as shown in the aforementioned study [16,17]. It is important to underline, in addition to the possible negative consequences of the disease, the positive aspects studied by several psychologists in the last 20 years, who have identified cancer as a possible source of growth, particularly thanks to the development of resilience in adolescent survivors [18]. Many scholars have discussed challenges associated with pediatric cancer and the need for evidence-based psychosocial interventions to support children, adolescents and their families [19].

Kazak and colleagues [20] identified seven key contributions that psychologists can make to collaborative and integrated care in pediatric cancer. These include managing procedural pain, nausea and other symptoms, understanding and reducing neuropsychological effects, treating children in the context of their families and other systems, applying an evolutionary perspective, identifying competency and vulnerability, integrating psychological knowledge into decision-making and other clinical care problems and facilitating the transition to palliative care and bereavement. The positive impact of clear and empathic communication of diagnostic and prognostic information as well as the use of digital games to distract children from their treatment process have been assessed [21,22]. Clinical studies have shown that psychosocial interventions are effective in reducing anxiety and depressive symptoms and improving the quality of life in pediatric oncology patients [23]. However, the disease can have a disruptive effect on family identity and structure, and families of pediatric cancer patients may experience negative outcomes. While some families adapt within twenty months of diagnosis, others continue to experience anxiety, depression, psychological distress and post-traumatic stress symptoms, even after the end of curative treatment. Therefore, evidence-based psychosocial interventions are crucial in providing support for pediatric cancer patients and their families [24,25].

At the same time, studies in the field highlight how the condition of illness also has a negative impact on the quality of life of parents [26,27]. A recent study [28] emphasizes how the relationship between mother and child changes during oncological treatments. Constantly caring for a sick child (caregiving burden) becomes a weight that generates stress that can be accentuated, among other things, by specific characteristics of the illness diagnosis and daily impediments that the child experiences. Anxiety, uncertainty, fear of the disease and its unpredictability and loneliness are just some of the most frequent emotional states that emerge [29,30]. The deterioration of the quality of life [31] depends, in fact, on the high level of stress experienced by parents [32], which can weaken the immune system to the point of developing post-traumatic stress disorder [32,33]. The well-being of parents can also depend on individual aspects, such as personality and the ability to positively reassess the situation [27] and derive satisfaction from their role as a parent, which amplifies in the course of illness. Further complications and worsening of the treatment process also arise from the consequences that the illness has on the child’s physical functioning, which affects their quality of life [34].

## 2. Materials and Methods

### 2.1. Research Aims

The aim of this research is to address a gap in the literature regarding the psychological experiences of young oncological patients and their parents. To achieve this, two interrelated objectives have been pursued.

The first objective is to investigate the experiences of parents whose children or adolescents have received an oncological diagnosis. Specifically, this involves exploring their emotions, experiences as caregivers and the impact of the disease on their lives since the diagnosis. Moreover, the focus is also on the coping strategies that parents have used and developed during their child’s hospitalization.

The second objective is to gain a deeper understanding of the experience of illness of young patients as there is a lack of systematic studies on the experiences of pediatric and adolescent patients. This general objective aims to contribute to making the relationship between patient–family–healthcare professionals more satisfying and, ultimately, to improve the success of the treatment itself. Starting from that, the study has specifically explored the emotions, dreams and fears of adolescents and children who have faced cancer in pediatric age. The study has tried to identify the needs and most recurring themes in their narratives as well as extract their experiences related to illness and their relationships with healthcare professionals, parents and peer groups.

### 2.2. Research Design

The study adopts a qualitative methodological approach, which frames individuals as active interpretative agents with their own points of view through which they construct their perspective on the world and the surrounding reality [35]. Qualitative methodology encompasses a heterogeneous variety of perspectives and traditions [36], but there is a general epistemological convergence in considering that its main strengths are the idiographic standpoint, which enables an in-depth analysis of the person within their context and a close examination of their unique perspective [37].

Therefore, semi-structured narrative interviews were used as a data collection method in order to capture the richness of participants’ narratives, leaving room for their unique storytelling [38] while maintaining a clear and pre-established structure [39]. Indeed, semi-structured interviews involve a set of previously constructed questions posed to the participants, with additional inquiries used to explore emerging topics and themes. It is a particularly flexible model, allowing for the collection of the details and richness of participants’ narratives [40] while maintaining a focus on the research aims [41]. Two different semi-structured protocols were adopted. Specifically, questions addressed to parents were, for example: “How did you feel when the physician communicated the bad news?”, “Did you feel understood from the healthcare personnel?”, “Have you noticed any changes in your daily life?”, “Did you feel that your role as a parent changed after the diagnosis?”. Questions addressed to adolescents were, for example: “How do you represent the illness to yourself?” (adolescent), “How do you feel in this new environment? Are there any spaces where you can play? How do you get along with other children?” (children), “Which people do you feel closest to right now? How would you describe the relationship you have with them?” (adolescent), “How do the treatments you receive make you feel? Do you feel listened to? What emotions do you experience most frequently?” (children), “What have been the most impactful changes in your lifestyle? How do you think you have dealt with or are dealing with these changes?” (adolescent), “If the illness were a journey, what would you fill your backpack with?”.

The verbatim transcriptions of the interviews were analyzed using the thematic analytical approach, which involves identifying themes through the analysis of narrative material, and through the support of the Atlas.ti software. The thematic analysis is a method of analysis which has been widely used for qualitative research [42] since it allows tackling different topics and is considered one of the most appropriate methodological strategies for studies that involve research questions and aims employing interpretative as well as explorative efforts [43]. As Braun and Clarke [44] explain, thematic analysis is functional if the following steps are followed: becoming familiar with the data (by reading it several times to identify possible patterns), generating initial codes (creating codes that summarize the main characteristics of the data), searching for themes (classifying codes into potential themes), reviewing themes (determining whether all the themes identified are relevant), defining and naming themes (creating a thematic map, identifying the heart of each theme) and, finally, producing the final written report [45].

Two analysts independently created codes and categories from the textual data. Then, they met with a third member to check the consistency and groundedness of their analyses, to solve eventual dissimilarities and to, finally, meet an accord for thematic generation. During the interview as well as the analysis phase, interviewers and analysts carefully checked for discrepancies and inconsistencies between patients’ and parents’ narratives. Indeed, patients and parents were part of the same family (even though not all the parents agreed to be interviewed—see below in Section 2.3) and it was important to understand if the illness was being experienced differently within the same family. However, no relevant discrepancy or inconsistency emerged (see below in Section 3), and therefore, there was no need to reanalyze data on that light.

### 2.3. Participants

The participants in the present study, parents as well as young patients, were recruited by a psychologist working at a hospital in Bari, located in the South of Italy, Puglia Region. Each participant signed an informed consent through which they expressed agreement to participate in a semi-structured narrative interview via telephone lasting approximately one hour as well as to an audio recording of the interviews.

With the regards to parents, the study included seven parents of adolescents with an oncological diagnosis. The group consists of seven parents, six of whom are women, and one is a man (see Appendix A). The age of the parents ranges from 36 to 58 years old (mean age of 47 years; standard deviation of 6.7 years). All participants are respectively the mother or father of the young patients (see above), and they are all married and living with their partners. Thus, all the parents interviewed are parents of some of the children/adolescents interviewed. All participants are Italians. Specifically, four of them live in the province of Bari (Puglia), one in the province of Brindisi (Puglia), and two live in Basilicata. Only one participant is currently employed in the telecommunications industry, while four women are on special leave or have closed their business (one is a teacher, one is an employee in a car dealership, one is a pharmacist and one is a pastry chef), and the other two women are housewives. The children of the interviewees included four males and two females, ranging in age from 11 to 17 years old (one boy is 11 years old, four are 16 years old and one is 17 years old; the mean age is 15 years, and the standard deviation is 2 years). The diagnoses are very varied, including: soft tissue sarcoma, testicular rhabdomyosarcoma, Ewing’s sarcoma, B-type acute lymphoblastic leukemia and Hodgkin’s lymphoma of bone origin (see Appendix B).

The study also included a group of children and adolescents with an oncological diagnosis, consisting of 5 females and 6 males. The age of the participants ranged from 7 to 18 years old (mean 15.1; standard deviation 2.96; mode of 17 years). Within the group, 10 participants were adolescents, and one was a child (using 12 years of age as a reference for entering adolescence). All participants live with their parents and were born in Italy. Most of them are residents of Puglia while some are from Basilicata. They are all followed by the pediatric onco-hematology department of a hospital in Bari. Their diagnoses are quite varied and include Ewing’s sarcoma, Hodgkin’s lymphoma, acute lymphoblastic leukemia, lymphatic leukemia, testicular sarcoma, osteosarcoma and rare sarcoma. At the time of diagnosis, they had a mean age of 13.64 years and a standard deviation of 3.17.

As stated below, not all parents of the 11 pediatric patients agreed to be interviewed, which explains the difference between the two samples.

The study followed the American Psychological Association Ethical Principles as well as the Declaration of Helsinki. Moreover, it was approved by the University of Pa-dova Ethics Committee (Ethical Code 86BC4C01AC73EE828B62F21440718EC9).

## 3. Results

The following section is divided into major themes emerging from parents’ and young patients’ interviews.

### 3.1. Parents’ Results

From the analysis of parents’ narratives, four major themes emerge.

#### 3.1.1. Emotional and Caregiver Burden

The emotions experienced by parents were at the heart of the interviews, allowing for a better understanding of the situation they have experienced. The uncertainties brought by the physical problems experienced by their children emerge, which led them to investigate the situation with pediatricians and specialists. In each of these cases, a diagnosis of oncological disease was reached, which many describe as impossible to forget.

“It seemed like an infection, but I had the feeling there was something more […] So when the diagnosis was given, I received it and didn’t receive it”.

(P2)

“[…] You can’t believe that something like this can happen to you. It really seems like you’re living in a movie”.

(P4)

“In the first few days, I felt like I was in a bubble, you can’t realize and understand what’s happening”.

(P7)

The act of “taking care”, which is crucial for a parent, becomes even more important in the case of a life-threatening illness in their child. From the experiences shared, confusion, pain and disbelief emerged, as seen before, which then turned into a fight that also involved the daily care of the child. The psychological burden of the disease overloaded the role of the parents, who were not physically ill but had to witness their child’s pain.

“When you see your daughter’s eyes imploring you to go home and not want to be sick anymore, that’s when it weighs on you, psychologically”.

(P5)

“You always have to pay a little attention, before you did not think about it, […] that is the thing that has changed more than anything else, you are always with that thought. Before you were more open, now you are much more worried, given his situation”.

(P3)

This experience of vicarious suffering made them feel the weight of caregiving as they did not know what would happen, were separated from their family and empathized with their child’s suffering, sometimes leading to a self-annulment due to totalizing care, feeling that they could only be “mothers”.

#### 3.1.2. Personal Growth

Alongside these extremely negative experiences, however, all parents have sought normalcy and shown their strength, their desire and need to fight the disease alongside their child, forcing themselves to accept the situation to move forward and fight. From this image, great experiences of resilience and positivity emerge, guided by a strong trust and hope towards the future and treatments. Some also indicate feeling fortunate compared to others regarding the diagnosis and having encountered positive aspects within the illness although they themselves specify that it was “difficult to say”.

“Every day was a victory, we fought this battle with a sword pointed towards the top, because we must reach the top, this is the goal”.

(P1)

At the same time, parents found the strength to move forward, without realizing where it came from. They did it for themselves and for their children, with whom, as some parents explain, they felt they had established a stronger relationship of mutual support and help in moments of discouragement while trying to remain the “same” parent.

“[…] it was a pleasure, I shared things with her, sometimes she did not tolerate me, but like every teenager. But in the end, it was a pleasure, for me certainly, to spend more time with her”.

(P6)

“In some sort of way, the shock helped us put things under a whole new perspective. You start appreciating small gestures, caresses, attention. […] And you realize how fundamental and important they are, the richness and satisfaction they can easily bring, if you let them touch you”.

(P7)

#### 3.1.3. Spirituality

Concerning spirituality, not intended as religiosity, some of the interviewed parents explained how their child’s illness changed their values, or those of the young patients. In the first case, a mother explained how the path after the diagnosis made her reassess her priorities, making her understand that some aspects she focused on before were superficial. Another interviewed woman explained how her older daughter’s illness opened the eyes of the young patient and her younger sister, allowing them to appreciate the little things and the time spent together as a family.

“At first, I suffered a little from this decision to leave work, but then it was an advantage from many points of view, because I took a break from some superficial things that I gave more importance to before, we changed our priorities a bit”.

(P6)

“We have all changed a bit: us, so to speak, but they changed a lot. I find myself with two different girls. On the one hand, it seems bad, but let’s say that it brought out the best in us. It put us in front of the true meanings of life”.

(P5)

Regarding religiosity, all the interviewees explained how it was of great help, an anchor to hold onto, and how prayer was a source of serenity and a request for well-being for the family. In addition, a mother explained how religiosity and prayer were ways to turn to Someone greater, surrendering to His will, guiding doctors in their work with her child.

“Through prayer and faith, I trust that God can give us the help we need, the strength”.

(P7)

“Completely surrender to God, ‘Jesus, you take care of it.’ I do not suggest the cure, because you are the doctor […], let Your will be done whatever it may be”.

(P1)

Religiosity led some parents to feel the closeness of God, His presence in their lives and in the journey of their children as if there was a powerful hand guiding the process. On the other hand, some were angry with God for what happened, feeling betrayed and questioning why it happened to their children and, in general, how there could be so much evil in the wards they frequented.

“[…] I felt a presence, or I wanted to think so, I felt it, praying I felt that things would go well and that there would be a higher power that would help my son”.

(P2)

Religiosity has helped a mother to believe that no matter how the treatments went, it would be fine because spirituality helps you think about life after death rather than the potential annihilation of life. The same woman provided a different perspective on prayer. She explained that she felt fear at the idea of going to church and praying because it would lead her to deeper introspection, bringing forth emotions that were too intense for her to confront at that moment.

“Faith helps you because it gives you hope. The thought that no matter how it turned out, it would be fine, because it helps you live death differently […] in the sense of a life after death. […] I’m almost afraid to enter a church because I don’t want to think too much. I’m afraid of thinking and reflecting too deeply. I’m afraid of where my thoughts might go, so I prefer to live without dwelling too much. I’m afraid of delving too deeply within myself and unleashing emotions that I can’t afford to deal with right now because I have to take it day by day, fight”.

### 3.2. Young Patients’ Results

From the analysis of young patients’ narrative, three major themes emerge.

#### 3.2.1. Emotions: From Confusion to Resilience

Initially, surprise, fear and anxiety arising from confusion in the new situation are described. There is a sense of injustice towards oneself and one’s family members who are also affected by this difficult journey. Uncertainty regarding the conditions, duration of treatment and future prospects is a recurring theme in the interviews.

“I thought I would never get out of it, that I did not deserve such a thing, that my family did not deserve it (although less often), that it is an unknown thing and that I only got to know it by experiencing it, but I would have liked to know something about it before. I think there is too much misinformation about it, before getting sick I never really wondered what it meant to be ill”.

(P9)

Fear is also reported in relation to the future and the possibility of a relapse. The hypothesis that the disease may reappear seems to be what scares patients the most. Another interesting aspect that emerged is the approach with technology and the search for information on the internet.

“I think that once everything is over, the problem could reappear. Last week I had a panic attack because, even though I had never read anything about the disease on the internet (also following the doctor’s advice), at that moment I searched [online] and saw that there was a possibility of a relapse. The idea of going through all of this again scares me. I have this fear a little bit”.

(P3)

However, most adolescents have shown great strength in contrast to these negative emotions, recounting and describing positive experiences. Despite finding themselves in a particularly difficult situation, many participants have managed to find a way to see the positive side of things, to appreciate even what they used to take for granted. Some even emphasize how they felt lucky in their misfortune, thanks to the people around them.

“I feel a myriad of different emotions, certainly at times after many moments that make me feel bad, like hospitalizations, I feel so much joy when I see my father and my sister again. Moments of joy are very frequent even though it doesn’t seem like it, and I always find a way to be as happy as possible”.

(P3)

#### 3.2.2. Relationships with Peers

It is interesting to note how the testimonies are particularly diverse regarding this topic: Some have not noticed any change with their lifelong friends; others struggle to meet and connect as they did before and have bonded more with people who are living with cancer and therefore share the burden of the disease and its treatment; while others have intensified their relationship with some and lost sight of other friends.

Those who are more positive report phrases full of gratitude and affection towards their companions, who show closeness despite the difficulty of meeting. These friends are mainly identified as “lifelong friends”.

“The relationship with my friends is beautiful, wonderful, special. I thank them so much […]. Even outside the hospital context, I have never felt different with them, there have been no changes”.

(P4)

An important aspect is understanding: According to what emerges from the narratives of adolescents, friends who found a way to communicate what was happening to the person directly involved were more aware and understanding towards them.

“In my relationship with my peers, there are ups and downs. Some moments have made me very happy, others not so much. At this age, not everyone understands what I am going through. I feel a little more understood by those who are maybe a little more mature, but not by others. Some of my friends know what I am going through but can’t bring themselves to contact me. Instead, they ask others for information... At first, it hurt me a little, and I thought they weren’t interested. Now I understand that we are not all made the same way, and I am not so upset about it. For example, one of my friends never contacts me, but when we see each other, he always pays attention to me. He wanted me to be there for his eighteenth birthday, even though I was in a wheelchair”.

(P3)

For those who mentioned it, the relationship with the boys and girls they met in the hospital who are going through the therapy journey with them seems to be particularly relevant.

“Apart from my mother, I feel very close to the people who are going through the same journey as me. Talking to P9 is different from talking to my best friend because she fully understands me”.

(P7)

#### 3.2.3. Personal Growth

From participants’ narratives, various aspects emerged relating to changes they faced during the illness and therapy. These changes were diverse, ranging from physical changes to changes in growth, thought, habits, and daily life. Initially, some of these changes were particularly difficult to accept, but acceptance and habituation gradually followed, thanks to the support of family and friends.

“I feel like I see life differently, that I have become aware of this environment. I am sure that I have to do things immediately (before I would not have expected to spend a month in the hospital, to have to shave my hair...)... At first, I dealt poorly with these changes, the hair loss was shocking. Now it seems like a good thing, I feel like I have something more than others that allows me to face everything”.

(P9)

The loss of independence is one of the most significant changes felt by the young people interviewed and is particularly distressing. Being unable, during adolescence, to move around freely, go out with friends, dress oneself, or take walks has had a great impact.

“I find it difficult because I cannot move around as much anymore, I am not as independent as before. I used to walk a lot, but now I can’t, I have to be more careful... Then, my social life has changed, I don’t physically go to school, I am no longer alone with myself... Before, I enjoyed taking a walk or being by myself even at home, now my parents won’t let me”.

(P3)

Daily life was shattered by the diagnosis, habits changed: Some feel a real fracture between their previous and current lives while others no longer recognize normality.

“I am living a completely different life, as if I entered another life, disconnected from the previous one. I have experienced physical changes, changes in my lifestyle, in my thinking... [The changes in thinking concern] how I dealt with the situation in February and how I am dealing with it now, even the little things. It seems to me that I am coping well, I make a lot of good resolutions, but they don’t always work out”.

(P7)

Some changes are also experienced as positive: The young people have noticed that they are living a path of growth, that they have acquired new important awareness of which they are proud.

“I have changed my character, my way of thinking. I see it as positive, I feel grown, changed. I like the changes I’m making at the character level. I do not like how I am using my time; I could do more useful things that would serve me better”.

(P8)

“I no longer have free time, I focus on what I like, I try to do the thing that happens more rarely […] I invest my time differently, I use every second. […] I feel that I am facing these changes positively, I have learned that time is not infinite and must be used responsibly. In the past [time] was infinite”.

(P11)

## 4. Discussion

The study aimed at investigating the experiences of parents whose children or adolescents have received an oncological diagnosis and at understanding the experience of illness of young patients. The results show that, while the oncological diagnosis triggers devasting feelings threatening daily experiences, with a profound emotional and caregiver burden, at the same time the disruption might open a new space of transformation and growth, where spirituality plays an important role. On the other hand, pediatric patients showed how ambiguous and fluctuating the oncological experience can be. The findings of the study overall indicate positive experiences for both parents and children, greater attachment to family and a deeper sense of self-awareness among the participants, which is consistent with prior research. As stated in Section 2.2 (see above), the analysts checked for inconsistency and discrepancy between patients’ and parents’ narratives whose existence would eventually be carefully analyzed separately. However, both parents and patients reported a great unity within the family group with regard to coping strategies and shared feeling, highlighting a common perspective emerging within individual and unique emotions. Indeed, the following paragraphs stress the importance of creating a cohesive familiar team in managing the illness as well as of safeguarding one’s own private moments.

The interviews reveal the weight of caregiving and its all-encompassing nature. Parents try to be constantly present, sometimes to the point of self-annulment, as explained in various studies [7,26,29,46,47]. Despite trying to maintain a sense of normalcy, parents often experience negative emotions of discomfort, fear and insecurity due to the unpredictability of the disease and its course, in line with what has been reported in the studies of Björk and colleagues [48]. Many other parents have reported having a good social support network and have been able to cope with the help of partners, family members and friends, who have assisted them in various tasks ranging from household chores, help with other children and financial support [49,50]. Financial problems arising from therapy are also significant, as reported by one interviewed mother. Despite these experiences, the interviews have shown the will to fight, to move forward and not to be defeated by the disease and daily difficulties that test both physical and mental strength. This need to fight leads to great experiences of resilience and growth, in line with what has been reported by Woodgate [51] and Van Schoors and colleagues [32], who emphasize the importance of family unity and parental couples, as reported by the interviewees, and how these aspects help to cope with the difficulties arising from the child’s illness. This personal growth involves a change in important values and a reprioritization of involved parents, demonstrating post-traumatic growth resulting from traumatic events, such as a cancer diagnosis, as reported in several studies [52,53]. This is accompanied by a strengthening of the relationship with the sick child and, in general, with the family, as emphasized by Picoraro and colleagues [54]. In the interviews, a mother defined the time spent with her daughter, despite the illness, as a pleasure, highlighting the satisfaction derived from care (compassion satisfaction), as reported in various research studies [55], and how this aspect of personal satisfaction in care leads to more positive experiences within the illness process. Regarding the aspect of anticipatory grief, anger and denial were reported by the parents involved in the interviews in the initial stages. It is hypothesized that they were going through the first two stages of the AG process theorized by Kübler-Ross. Regarding the moment of diagnosis, all parents have a vivid memory, as reported by Parker and Johnston [56]. From the beginning, the doctor-patient relationship is characterized by clarity, trust and empathy, with parents expressing satisfaction with the care and relationship established with the healthcare staff, which has been reported in some literature. After the diagnosis, the referring oncologist always took care to speak personally with the young patients, with great tact but clearly and realistically about the diagnosis, treatment path and the possible outcomes.

Finally, regarding the aspect of spirituality, it emerges from the literature [56,57,58,59] how it helps as an anchor to understand and make sense of the illness, to move forward and provide hope and comfort, acting as a guide in uncertainty for parents, as emphasized by the interviewees. This support is made possible primarily through prayer, but also through contact with sacred objects, as explained by the interviewees and studies by Rossato and colleagues [60,61]. As emphasized by the parents, spirituality has helped them feel connected to a higher power and to seek health and assistance from God, in line with the findings from the previous study [60]. Throughout the interviews, the centrality of support, spirituality, hope and positivity emerges as coping strategies to face the situation.

The adolescents reported a range of emotions and fear of relapse, and the re-experiencing of pain associated with treatment characterized their experiences. Changes in physical appearance, such as hair loss, had a significant impact on the adolescents’ self-perception and sense of identity, contributing to feelings of inferiority and a struggle to recognize themselves. The aggressive cycles of therapy led to a partial loss of independence, and the participants reported significant discomfort in relying on family for even the simplest tasks. However, some participants experienced growth and development of awareness and coping skills as a result of their illness. The analysis highlights the complexity of the experiences of adolescents with cancer and calls for a more nuanced understanding of the factors that contribute to their quality of life.

However, since the pediatric population under study falls within the adolescent phase, it is necessary to highlight some characteristics of individuals in this age group. Firstly, it is clear that adolescents have different needs compared to children and adults diagnosed with the same condition. Adolescence is an extremely delicate phase in a person’s growth and development, characterized by specific developmental tasks. It is a phase of biological, social and psychological changes that lead individuals to feel the need to discover, explore and define their own physical and psychological identity [62,63]. For this reason, when they feel uncertain about the potential progression of the disease and perceive a lack of necessary information to understand how to cope with it, they tend to experience increased levels of stress, anxiety, depression and isolation [64]. This is not the case if they feel aware of what is happening to them, which is why it is common for them to express a desire to participate in decisions regarding their treatment paths within the first year after the illness [65,66]. It is important, therefore, to understand the decision-making process in adolescents and find the best way to involve them in decisions regarding the treatment of their condition, in order to avoid increasing or prolonging their suffering by applying principles that do not align with their needs [67]. A factor that reinforces the desire to actively participate in treatment decisions is the sense of self-efficacy, which refers to the ability to feel capable in the face of challenges that they encounter. This feeling is common among many adolescents [66].

## 5. Conclusions

From these interviews and parents’ accounts, we gain a more precise understanding of the experiences of caregivers of adolescents with oncological diagnoses. Considering what has emerged from the research, there is the need to design and implement future projects aimed at supporting the entire family. This will allow for more accurate and patient- and family-centered interventions, not only to support families but also to provide new insights for possible future research on the experiences of parents and young patients, coping strategies, psychological support or techniques that have helped the entire family to cope, such as pet therapy or art therapies, which were not proposed in this study. Specifically, it would be interesting to expand beyond the nuclear family and include the extended family, encompassing individuals indirectly affected by the experience of illness. This would provide insight into how relatives and friends contribute to supporting the illness experience. Additionally, it would be valuable to replicate the interviews with the same individuals at various stages of the illness and treatment in order to achieve a more comprehensive understanding of the overall illness experience. In this regard, further exploration of the lived experiences associated with the moment of diagnosis would be worthwhile, with particular emphasis on the methods employed to convey the difficult news. This would also facilitate the evaluation of the contribution of psychological support provided by hospitals and aid in its implementation.

From this recruitment process, a group of 18 participants was obtained. This is a limited group, and therefore the narratives cannot be generalized. However, it has provided very interesting indications for further research through the involvement of other patients, particularly pediatric patients. Despite these limitations, the present study has highlighted how in the interviewed young patients, certain central themes consistently emerge, which families and therapists should carefully consider. Among these, particularly relevant are: the numerous and diverse changes that adolescents and children affected by oncological diseases must face, ranging from physical changes to changes in habits, self-perception, use of time and level of independence; the relationship with peers, sometimes seen as the main source of support, other times fraught with difficulties due to the lack of understanding and yet again rediscovered in people known in the hospital who are undergoing treatment and care for cancer; the multiple emotions experienced during the course of therapy, ranging from astonishment at the time of diagnosis, uncertainty, fear, anger and frustration during treatment, to joy and happiness with approaching discharge, still punctuated for some by anxiety at the thought of relapse; the relationship with healthcare personnel, evaluated as particularly relevant and positive; the perceived support, especially from closest family members, which allows patients to feel accompanied on this tortuous journey.

Finally, these results can foster interesting insights for enhancing clinical care services. Firstly, as previous studies have highlighted [37,68], in times of traumatic events, a more structured social support can be of utmost importance. When facing the attempt to find a meaning, self-help groups can be very powerful, also working as an important emotional stabilizing tool through correspondence and sharing [69]. Thus, clinical institutions could provide more opportunity for mutual aid. At the same time, results show that it is important to provide clinical intervention, to both parents and pediatric patients, that help in maintaining a sense of continuity with ordinary life, thus avoiding the risk of creating a spiral of sufferance which can result in an emotional burden and that can make nurturing interpersonal relationships difficult.

## Data Availability

The data presented in this study are available on request from the corresponding author. The data are not publicly available due to privacy.

## Appendix A

Brief description of parents taking part in the research.

Participant 1: A 43-year-old woman who lives in the province of Bari, Italy. She is a mother of three boys and a French teacher who had to request a special leave to take care of her son during his treatments. She has been married for several years and shares a strong bond of love with her husband, accompanied by a strong faith in God. Her 11-year-old son is being treated for soft tissue sarcoma in the pediatric onco-hematology department. At the time of the interview, he is undergoing his last cycle of chemotherapy. According to the mother, he has always shown great maturity in dealing with his illness.

Participant 2: A 53-year-old woman living in Bari. She is a mother of three children, two adult daughters and a 17-year-old son with a diagnosis of rhabdomyosarcoma of the right testicle, who at the time of the interview is in the follow-up phase. She is a homemaker, married for many years and considers herself religious.

Participant 3: A 58-year-old man residing in Bari, he is the father of three children. He identifies himself as a religious person and works in the telecommunications field.

Participant 4: A 45-year-old woman residing in the province of Bari. She is a homemaker and mother of three children, two boys and a girl. She identifies herself as a religious person and has been married for several years. Her eldest child, a 16-year-old boy, is undergoing treatment at the onco-hematology department in Bari for a recurrence of Ewing’s sarcoma in the lungs, following a diagnosis of Ewing’s sarcoma in the left tibia five years prior.

Participant 5: A 48-year-old woman residing in the province of Brindisi. She is mother of two daughters and employed at a car dealership. She has requested exceptional leave to care for her eldest daughter (16 years old) who has been diagnosed with B-cell acute lymphoblastic leukemia. She is married and shares a strong bond with her husband. She identifies herself as a religious person and her daughter is currently undergoing the final cycle of chemotherapy.

Participant 6: A 51-year-old woman residing in Matera, mother of three children and a pharmacist. She has taken leave from work to care for her daughter, after initially alternating with her husband for the care. She is married and identifies herself as a religious person. Her daughter has been diagnosed with Hodgkin’s lymphoma of bone origin.

Participant 7: A 36-year-old woman residing in Basilicata, married with two children, a 16-year-old boy and a 4-year-old girl, is a pastry chef who had to close her business to accompany her eldest son, who has been diagnosed with B-cell acute lymphoblastic.

## Appendix B

P8: A 7-year-old boy diagnosed with acute lymphoblastic leukemia in July 2021, when he was 6 years old. At the time of the interview, he was undergoing therapy. He has formed an intense bond with two other children who have undergone therapy with him. He has suffered greatly from the loss of one of them.

P9: A 13-year-old boy diagnosed with a rare soft tissue sarcoma in November 2021. After several cycles of chemotherapy, he underwent surgery. At the time of the interview, he was undergoing therapy in its final cycles. He is the eldest of three siblings and lives in the province of Bari with his family.

P10: A 17-year-old girl who has been diagnosed with osteosarcoma and has been undergoing treatment since February 2022. She has already undergone surgery and she is in the final cycles of treatment. She attends high school.

P11: A 17-year-old boy who was diagnosed with testicular sarcoma in January 2021. He completed treatment in June 2022, and he is currently in the follow-up phase. He has two older sisters and resides with his family in Bari.

P12: A 18-year-old girl who has been diagnosed with lymphatic leukemia. She received the diagnosis in April 2022 and has been undergoing treatment since then.

P13: A 17-year-old boy who was diagnosed with acute lymphoblastic leukemia type B in May 2022 and is still undergoing treatment. He lives with his parents and a younger sister in Basilicata.

P14: A 16-year-old girl who was diagnosed with acute lymphoblastic leukemia in February 2022. She has completed the first cycle of treatment and is currently undergoing the second cycle at the time of the interview. She is very focused on the future, the end of treatment and returning to “normal” life.

P15: A 12-year-old boy with lymphatic leukemia who received the diagnosis in March 2022 and has been undergoing treatment since then.

P16: A 16-year-old girl who was diagnosed with acute lymphoblastic leukemia type B in December 2021. She has been undergoing treatment since then. She is currently facing the last cycle of chemotherapy at the time of the interview. She has a younger sister, and they live with their family in Brindisi.

P17: A 16-year-old girl who has a rare form of Hodgkin’s lymphoma originating from the bone. She started treatment in November 2021 and completed it in April 2022. She is the oldest of three siblings and lives with her family in Matera, Basilicata. At the time of the interview, she was in the follow-up phase.

P18: A 17-year-old boy who was readmitted to the hospital in May 2022 for a relapse of Ewing’s sarcoma in his left tibia, which was first diagnosed five years ago in 2017. At the time of the interview, he had completed the first five cycles of chemotherapy and was waiting for surgery. He has a younger brother and sister, and they all live with their parents in the province of Bari.
